# Onset of two-dimensional superconductivity in space charge doped few-layer molybdenum disulfide

**DOI:** 10.1038/ncomms9826

**Published:** 2015-11-03

**Authors:** Johan Biscaras, Zhesheng Chen, Andrea Paradisi, Abhay Shukla

**Affiliations:** 1 Institut de Minéralogie, de Physique des Matériaux et de Cosmochimie (IMPMC), Sorbonne Universités—UPMC Univ. Paris 06, UMR CNRS 7590, Muséum National d'Histoire Naturelle, IRD UMR 206, 4 Place Jussieu, 75005 Paris, France

## Abstract

Atomically thin films of layered materials such as molybdenum disulfide (MoS_2_) are of growing interest for the study of phase transitions in two-dimensions through electrostatic doping. Electrostatic doping techniques giving access to high carrier densities are needed to achieve such phase transitions. Here we develop a method of electrostatic doping which allows us to reach a maximum *n*-doping density of 4 × 10^14^ cm^−2^ in few-layer MoS_2_ on glass substrates. With increasing carrier density we first induce an insulator to metal transition and subsequently an incomplete metal to superconductor transition in MoS_2_ with critical temperature ≈10 K. Contrary to earlier reports, after the onset of superconductivity, the superconducting transition temperature does not depend on the carrier density. Our doping method and the results we obtain in MoS_2_ for samples as thin as bilayers indicates the potential of this approach.

## Abstract (web summary)

Inducing phase transitions in atomically thin films of layered materials requires efficient electrostatic doping techniques to reach high carrier densities. Here, the authors present a doping technique which induces superconductivity by reaching high *n*-doping density in few-layered MoS_2_ on glass substrates.

## Graphical abstract

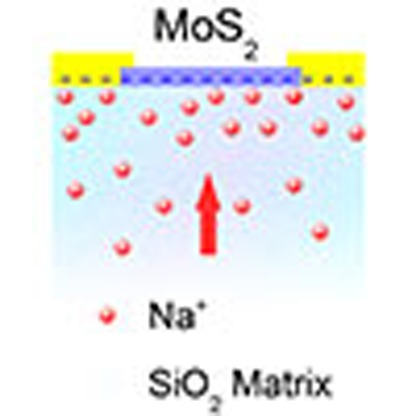

Layered materials are being extensively studied for their exceptional properties when reduced to few atomic layers. The promise of producing innovative electronic devices is alluring. So is the perspective of electrostatic doping to induce substantial carrier density and phase transitions^1^ in two-dimensions (2D). Recent research in both fields has widely used molybdenum disulphide (MoS_2_) as a prototype.

Bulk MoS_2_ is a semiconductor with an indirect electronic band-gap. In the limit of the single layer a direct gap semiconductor is obtained^2^,^3^ as the indirect transition energy becomes larger than the direct gap at the *K*, *K′* points of the Brillouin Zone. The strong light–matter interaction at these points^4^ and the lifting of the spin degeneracy of the valence and conduction bands due to inversion symmetry breaking make single layer MoS_2_ an excellent material for transistors in electronics, opto-electronics and the developing valleytronics field^5^,^6^,^7^,^8^.

Chemical intercalation can induce an *n*-doped metallic and superconducting state^9^,^10^, a fact that has inspired electrostatic and electrochemical doping of few-layer MoS_2_. The insulator—metal transition and carrier densities of about 10^13^ cm^−2^ have been reported using solid dielectrics like SiO_2_ (refs 11, 12) and HfO_2_ (ref. 13). Using an ionic liquid as a dielectric, densities above 10^14^ cm^−2^ could be obtained with superconductivity appearing in a narrow region between 8 × 10^13^ and 3 × 10^14^ cm^−2^ and a maximum critical temperature of 11 K (refs 14, 15). Both these techniques involve delicate device issues and whereas a solid dielectric does not allow for very high doping, the liquid gating method requires great care to avoid possible intercalation, electrochemical reactions^16^ and strain during the liquid to solid transformation of the dielectric, which may be avoided by using a solid ionic conductor^17^.

Here we develop a method of electrostatic doping that allows us to reach a maximum *n*-doping density of 4 × 10^14^ cm^−2^ in the simplest of devices consisting of few-layer MoS_2_ on glass. We produce few-layer MoS_2_ samples with our anodic bonding method^18^,^19^,^20^ that uses a glass substrate containing mobile ionic species at high temperatures (≈400 K or higher). A layered sample is placed on the substrate and an electric field induces an ionic drift and a space charge at the sample-substrate interface leading to a very strong electrostatic field. The electric field ‘sticks' the layered sample to the glass substrate and mechanical exfoliation of the attached sample leaves few-layer residue on the glass substrate. Our ‘space charge doping' technique^21^ is a natural extension of this method for which we start with the few-layer sample lying on the glass substrate (either soda-lime glass or borosilicate glass). At high temperature, the glass substrate can be seen as a negatively charged static matrix (mainly SiO_2_ units) with mobile positively charged ionic species depending on the glass composition, in our case Na^+^ ions^22^. Applying a positive (resp. negative) voltage on the gate electrode at the back of the substrate generates a drift current of mobile Na^+^ going towards (resp. away from) the sample and creating an accumulation (resp. depletion) layer below the interface as shown in Fig. 1. This space charge at the interface induces electrostatic doping of the few-layer sample. Mobility of Na^+^ in glass in our experimental conditions remains small, which means that charging can be finely controlled and measured in real time over minutes to hours. Thus, by applying a positive or negative gate voltage of the order of 1–100 V, the sample can be doped ambivalently with a consequent variation of its resistivity, which can be monitored along with the gate current as a function of time. The doping process can be quenched at a particular value by cooling the sample to room temperature or below, thus ‘freezing' the Na^+^ ions in the glass matrix and annulling the ionic mobility and drift current. The carrier density that depends on the total charge transferred during the doping process is determined in this static state by Hall effect measurements. Heating of the polarized substrate causes a drift current which tends to nullify the space charge layer and thus remove electrostatic doping of the sample. This process can be accelerated by the application of an appropriate electric field. The technique allows to dope/un-dope the samples multiple times with consistent results, even after *ex-situ* storage. Raman spectra of samples before and after doping cycles do not show any signs of modification (Supplementary Fig. 1 and Supplementary Note 1) though returning to an un-doped state after very high doping (>10^14^ cm^−2^) can sometimes result in an increase in residual resistivity possibly due to mechanical strain in the sample. In this study, we show the result of space charge doping applied to few-layer MoS_2_ with varying thickness. An insulator–metal transition is induced at moderate doping. The very high carrier density accessible with our doping technique allows us to subsequently induce superconductivity.

## Results

### Sample characterization

MoS_2_ samples are prepared and contacted as described in the Methods section. As prepared samples typically show resistance of the order of GΩ in ambient conditions. Transport measurements are performed in a high vacuum cryostat coupled to an external 2-T electromagnet for Hall measurements. After annealing in the cryostat at 420 K for several hours the resistance decreases to 20–100 kΩ. The properties of MoS_2_ have been shown to be particularly sensitive to adsorbates, especially in the few-layer limit^23^,^24^, explaining this observation. The annealing also ensures the removal of any residual space charge from the sample fabrication step. The initial *n*-doping density is found to be around 10^13^ cm^−2^. This substantial initial doping originates from shallow Na^+^ donor impurity levels due to interfacial ions^25^ as we show below.

### Insulator–Metal transition

**Figure 2:**
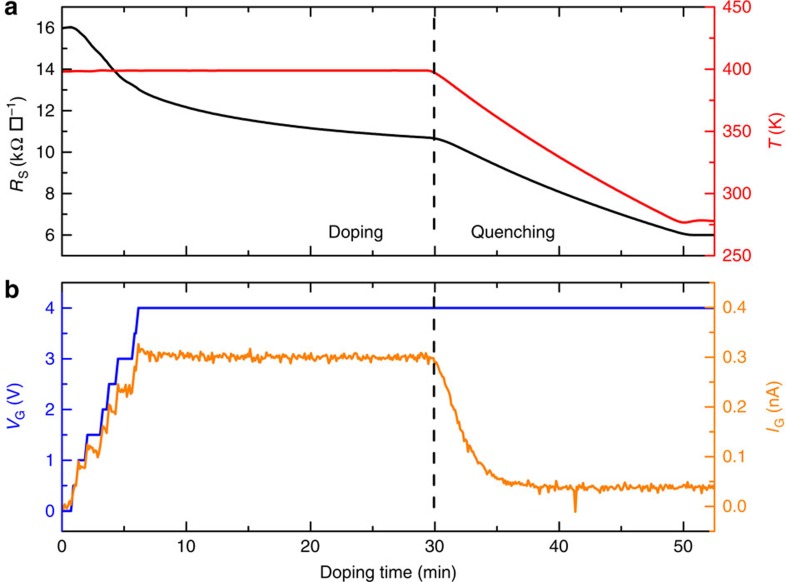
Space charge doping dynamics. (**a**) Sheet resistivity (black line, left axis) of a 2-nm thick sample as a function of doping time during space charge doping experiment with positive gate voltage at *T*=400 K and the subsequent quenching in temperature. The temperature is shown as the red curve (right axis), the vertical dash line marks the limit between the doping itself and the following temperature quench. (**b**) Applied gate voltage (blue line, left axis) and gate current measured (orange line, right axis) during the space charge doping experiment.

**Figure 3:**
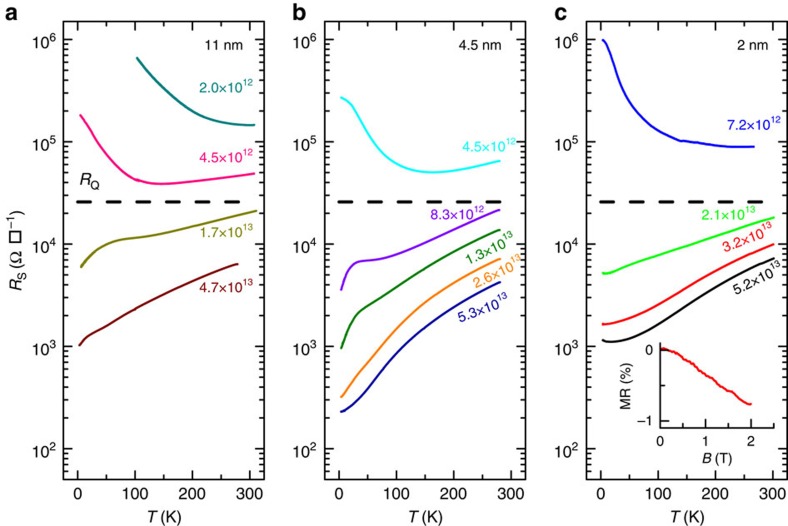
Metal–Insulator transition. (**a**–**c**), Sheet resistance as a function of temperature of the three MoS_2_ samples for low to moderate doping levels achieved with space charge doping. The carrier densities measured at room temperature are displayed next to each curve (in units of cm^−2^). The samples (11, 4.5 and 2 nm) are labelled by thickness as measured by AFM. The horizontal dashed line is the quantum of resistance *R*_Q_=*h*/*e*^2^. The inset of panel **c** shows the magnetoresistance MR=Δ*R*/*R* of the 2 nm sample as a function of perpendicular magnetic field at 4.7 K during the doping experiment corresponding to the red curve of the main panel.

We show results from three samples with varying thickness: 2 nm (bilayer according to Raman measurements), 4.5 nm (∼7 layer) and 11 nm (∼17 layer). An example of the space charge doping procedure is shown in Fig. 2. This procedure involves doping the sample at high temperature (≈400 K) by application of a gate voltage during a time determined by the desired change in resistivity, quenching to room temperature followed by resistivity and Hall measurements as a function of temperature down to ∼3 K. The insulator–metal transition occurring in the low to moderate carrier density range (0.3–5 × 10^13^ cm^−2^) is clearly visible for all samples, as shown in Fig. 3. As the doping dynamics is slow and electrostatic equilibrium is never reached in our experiments (Fig. 2), the absolute value of the gate voltage used during the polarization is not relevant. Indeed, the same carrier density can be obtained with a lower gate voltage but longer time, as what counts is the accumulated space charge. The metallic state is reached by applying a positive gate voltage (*n*-doping), the insulator–metal transition (indicated by a horizontal dashed line in the figure) always occurring around the quantum of resistance (*R*_Q_=*h*/*e*^2^) despite differences in mobility and number of layers, consistent with previous reports^15^. This transition corresponds to the Mott–Ioffe–Regel limit^26^ in 2D when the electronic wavelength is of the same order of magnitude as the mean free path, that is *k*_F_*l*∼1.

It is worth noting that low temperature localization behaviour (∂*R*/∂*T*<0, refs 27, 28) can be seen on the metallic side of the metal–insulator transition. In the inset of Fig. 3c we show an example of such localization for the 2 nm sample at a doping of 2 × 10^13^ cm^−2^ and at 4.7 K through the measurement of negative magnetoresistance. This behaviour originates from quantum interference corrections to the conductivity (electrons are back-scattered coherently after multiple collisions) and thus is only seen at low temperature, while the metal–insulator transition corresponding to the quantum of resistance originates from strong localization of electronic wave-functions (the mean free path is shorter than the electronic wavelength and the electrons do not propagate). It is thus seen at much higher temperatures (300 K).

The transition can be reversed by hole doping (with a negative gate voltage) which pushes the sample back into the insulating state. Low temperature *I*−*V* characteristics in the insulating state show non-Ohmic behaviour typical of variable range hopping (Supplementary Figs 2 and 3 and Supplementary Note 2).

**Figure 4:**
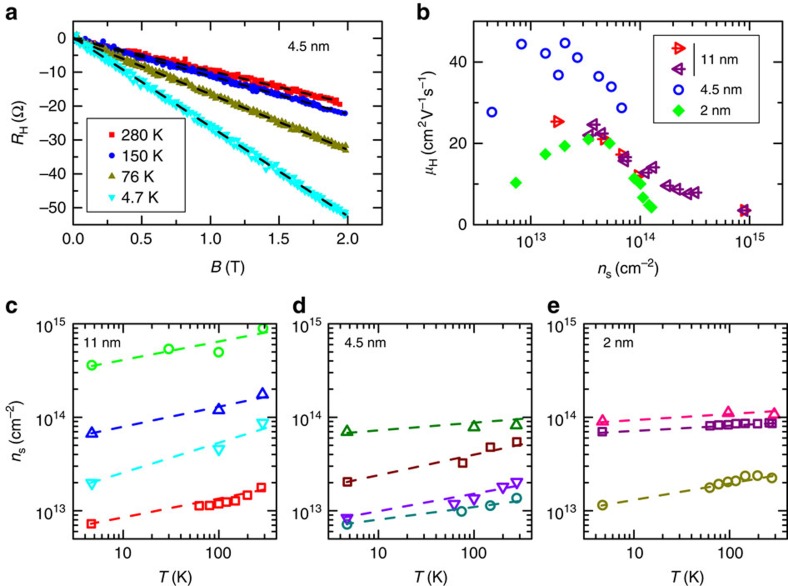
Hall effect. (**a**) Anti-symmetrized Hall resistance as a function of magnetic field of the 4.5 nm sample measured at a space charge induced carrier density of ∼2 to 5 × 10^13^ cm^−2^ at different temperatures, showing a decrease in carrier density at low temperatures due to impurity level trapping. Black dashed lines are linear fits. (**b**) Hall mobility of the three samples measured at 280 K as a function of the space charge induced carrier density showing an increase with doping at low density and decrease at high carrier density. For the 11 nm sample the red arrows pointing to the right are measurements made while increasing the carrier density, while the purple arrows pointing to the left are made while decreasing carrier density after maximum doping. (**c**,**d**,**e**), Carrier density variation with temperature at some fixed doping levels for each sample. Dashed lines are guides for the eye.

The carrier densities are measured by Hall effect. All the measured Hall resistances *R*_H_(*B*) show linear behaviour with the magnetic field in the range we used (Fig. 4a) which allows to determine the Hall carrier density through the formula *R*_H_/*B*=1/*en*_S_. The carrier concentration decreases with temperature as shown in Fig. 4c–e. The cation mobility in the glass being negligible below 300 K, this effect is not due to a loss of polarization in the glass and has been verified to depend only on temperature and not on applied gate voltage. This can be attributed to the formation of shallow donor impurity levels in the gap near the bottom of the conduction bands by the Na^+^ ions at the glass surface^25^. These traps can capture electrons from the MoS_2_ sample at low temperature, thus reducing the carrier density, while at high temperatures they are ionized resulting in more efficient space charge doping. Carrier densities are extracted directly from the Hall coefficient assuming a single band with isotropic mass and diffusion coefficient. High doping could involve carriers in multiple valleys with different band mass because of the complex structure of the MoS_2_ conduction band^15^. However, recent *ab-initio* band calculations^29^ show that for electrostatically *n*-doped MoS_2_ the one band Hall constant formula holds even beyond carrier densities of 10^14^ cm^−2^, with small variations (20%) at 4 × 10^14^ cm^−2^. A simple model assuming two bands implies non-linear Hall resistance behaviour not seen in our measurements.

The mobilities derived from the Hall carrier density at high temperature (280 K) are shown in Fig. 4b. At low carrier density, the mobility increases with carrier density as it has been reported in solid states FET devices^12^, whereas the high density mobility is limited by increased scattering processes^30^,^31^,^32^. There is also the possibility that the electrostatic pressure at very high doping may locally deform the crystal lattice to adapt to the roughness of the amorphous glass substrate. Indeed at densities ∼10^14^ cm^−2^ we can estimate that the electrostatic pressure reaches 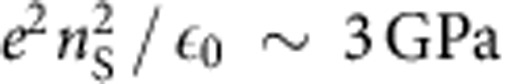 that is significantly smaller but not negligible compared to the Young's modulus of ∼0.3 TPa and breaking stress of ∼20 GPa of monolayer MoS_2_ (ref. 33). However, MoS_2_ nanosheets can be deformed reversibly to several nanometres parallel to *c* axis^34^, while the roughness of our substrates has been measured to be between 0.2 and 0.8 nm through atomic force microscopy (AFM). Thus the possibility of irreversible deformation is small. Accordingly, mobility before and after maximum doping for the 11 nm sample does not change as shown in Fig. 4b, however, irreversible deformation in the 2 nm sample may be possible at very high doping.

At low temperatures (4.7 K) the mobilities expectedly increase enormously due to reduced phonon scattering. The 2 nm sample showed mobilities from 80 to 200 cm^2^ V^−1^ s^−1^ for low to moderate (∼10^13^ cm^−2^) doping. The thicker 11 nm sample had mobilities from 200 to 700 cm^2^ V^−1^ s^−1^ in the same doping range. The intermediate 4.5 nm sample displayed high mobilities up to 1,800 cm^2^ V^−1^ s^−1^ at low doping, decreasing to ∼1,300 cm^2^  V^−1^ s^−1^ at 2 × 10^13^ cm^−2^. These values are in agreement with previous reports on MoS_2_ in these doping regimes^35^,^36^. The differences in mobilities in the three samples are most likely related to the quality of the initial bulk samples.

### Superconductivity

Beyond the insulator to metallic transition which occurs at moderate doping, the samples were doped to higher carrier densities. The maximum carrier densities measured at room temperature were 1.4 × 10^14^ cm^−2^ for the 2 nm sample, 7.8 × 10^13^ cm^−2^ for the 4.5 nm sample and 8.9 × 10^14^ cm^−2^ for the 11 nm sample. The much higher value obtained for the 11 nm sample is explained by the fact that it is placed on glass with a higher sodium concentration (soda-lime instead of borosilicate glass). At the relatively low maximum carrier density achieved in the 4.5 nm sample no superconducting transition was found.

**Figure 5:**
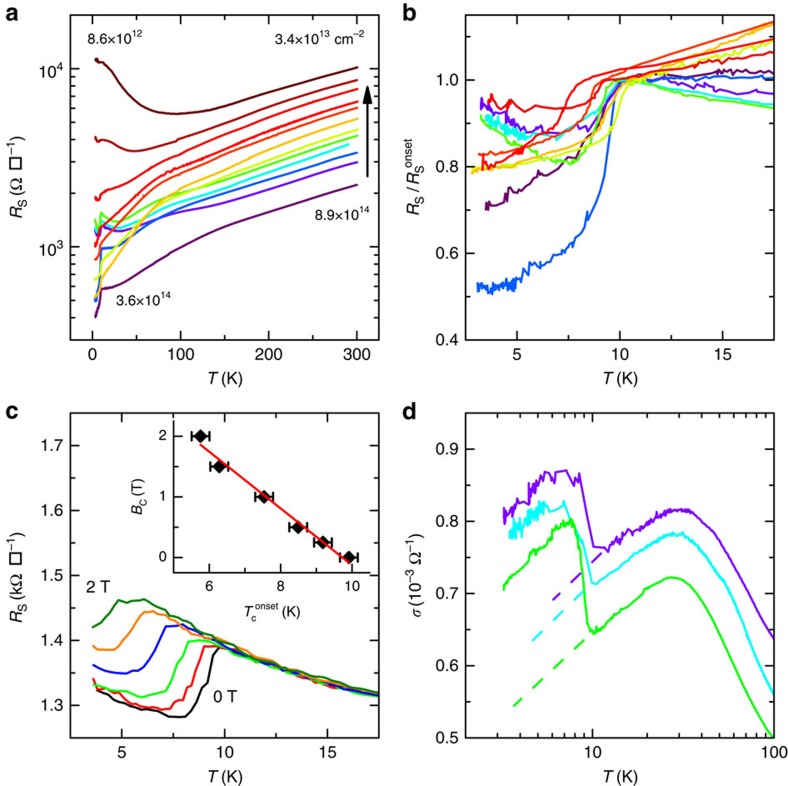
High doping regime of 11 nm sample. (**a**), Sheet resistance of the 11 nm sample as a function of temperature for high doping levels. The arrow indicates the sequence of measurements. The carrier densities displayed (in units of cm^−2^) are measured by Hall effect at room temperature and 4.7 K for the two extremal curves, showing the range of measurements. (**b**), Sheet resistance normalized by its onset value as a function of temperature showing incomplete superconductivity. (**c**), Sheet resistance as a function of temperature under perpendicular magnetic field (0, 0.25, 0.5, 1, 1.5 and 2 T) at fixed carrier density (∼2 × 10^13^ cm^−2^). Inset: Critical field *B*_C_ as a function of onset critical temperature, with linear fit (red line). The error bars relate to the extraction of the onset critical temperature. (**d**), Weak localization on both sides of the superconducting transition at some doping densities extracted from panel **b**.

Figure 5 shows the measurements on the 11 nm sample that was first doped to the maximum carrier density where a sharp fall in resistance at low temperatures, indicating superconductivity, appeared. Doping was then progressively decreased resulting in the expected monotonic increase in the room temperature sheet resistance and eventual disappearance of superconductivity as shown in Fig. 5a for the whole temperature range. The low temperature sheet resistance evolves in a more complicated manner with decreasing doping. Possible weak localization below 20 K is seen (∂*R*/∂*T*<0) coexisting with superconductivity though it disappears at still lower doping. Fig. 5b shows resistivity curves normalized to the superconducting onset temperature in the range up to 20 K. The transition temperature is independent of the doping despite the wide range of measured carrier densities (from ∼10^13^ to 3 × 10^14^ cm^−2^) and roughly constant at ≈10 K. A signature of the superconducting transition is its dependence on the applied perpendicular magnetic field. As shown in Fig. 5c, the transition shifts to lower temperatures with an increasing perpendicular magnetic field. This well known variation of the transition temperature is approximately linear close to *T*_C_ as is observed for the onset superconducting temperature in our sample in the inset of Fig. 5c. By fitting our data to the Bardeen-Cooper-Schrieffer (BCS) formula for this variation we estimate the zero Kelvin critical field to be *B*_C_(0)∼3 T.

**Figure 6:**
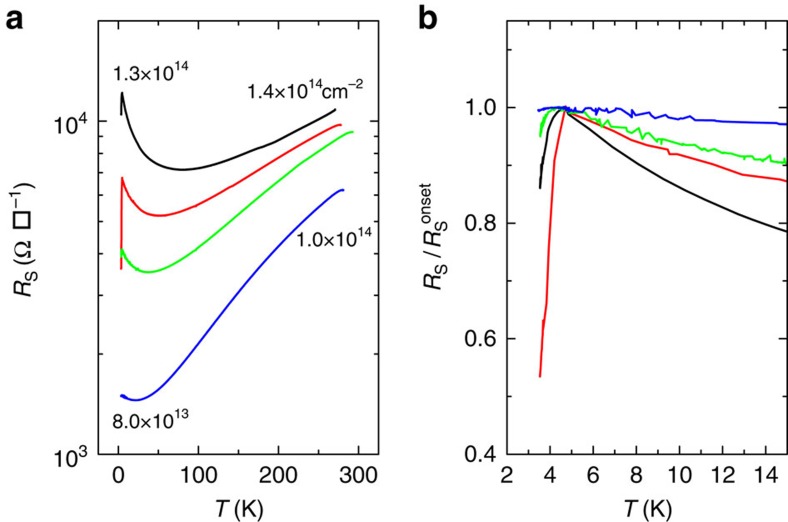
High doping regime of the 2 nm sample. (**a**), Sheet resistance of the 2 nm sample as a function of temperature for high doping levels. The carrier densities displayed (in units of cm^−2^) are measured by Hall effect at room temperature and 4.7 K for the two extremal curves. (**b**) Sheet resistance normalized by its onset value as a function of temperature showing incomplete superconductivity.

As shown in Fig. 6a the 2 nm bilayer was doped progressively from low to high carrier concentrations which provoked both an increase in resistance and eventually a superconducting transition. The increase in sheet resistance at the highest doping levels is probably caused by irreversible crystalline deformation in this ultra-thin sample as discussed above. Again, below 20 K signs of localization (∂*R*/∂*T*<0) coexist with superconductivity. Despite the low *T*_C_≈4 K in this sample the onset of the transition is clear.

Both samples thus display an incomplete low temperature superconducting transition with significant differences from the one reported in ref. 15.

First, it is incomplete with non-zero resistance below *T*_C_ as can be seen in Fig. 5 and similar to reports in refs 14, 16 . This implies the existence of a dissipating normal phase at temperatures lower than *T*_C_ (defined as the superconducting onset temperature).

**Figure 7:**
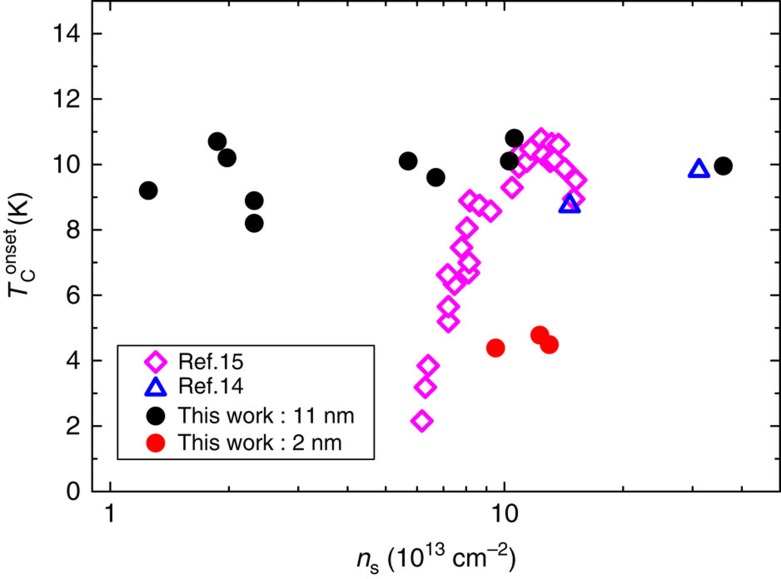
Superconducting critical temperature. Critical temperature corresponding to the onset of the superconducting transition as a function of Hall carrier density measured at low temperature for the 2 nm (red circles) and the 11 nm (black circles) samples. Open symbols represent values reported in ref. 15 (pink open diamonds) and ref. 14 (blue open triangles) and are added for comparison.

Second, each sample shows this behaviour over doping ranges that are different from those reported earlier. The 11 nm sample shows a doping independent ∼10 K critical temperature over a large carrier concentration range, from a strikingly low 1.3 × 10^13^ (at which neither 2 nm nor 4.5 nm samples are superconducting) to 3.6 × 10^14^ cm^−2^(Fig. 7). The bilayer sample shows a lower *T*_C_≈4 K from carrier concentrations of 9 × 10^13^ to 1.4 × 10^14^ cm^−2^. Though electrostatic doping is probably confined to a thickness corresponding to less than the bilayer in both samples, the higher critical temperature in the thicker sample could be due to a *c* axis superconducting coherence length in superconducting MoS_2_ that is greater than the bilayer thickness. Even in the insulating regime the conductivity shows a transition from 2D to 3D behaviour in going from the bilayer to the thick sample (Supplementary Note 2).

Third, in some cases superconductivity is preceded by non-metallic behaviour with increase in resistance as temperature decreases (∂*R*/∂*T*<0), characteristic of localization. As shown in Fig. 5d this localization corresponds to a logarithmic decrease of conductivity with temperature corresponding to 2D electrical transport^27^,^28^. Residual fermions continue to localize below *T*_C_.

As a cross-check on the reproducibility of our results all three samples were measured at least twice. Between measurements each sample was reduced to its original post-annealing doping state and subsequently removed from the cryostat, measured with Raman spectroscopy and stored in a primary vacuum for days or weeks. Reintroduction into the cryostat followed by doping cycles and measurement gave reproducible results.

## Discussion

Non-zero resistance in superconducting materials is an ongoing matter of research as multiple factors could lead to the appearance of dissipation above and below the superconducting transition, especially in low dimensions. Indeed, thermal vortex generation produces dissipation at any non-zero temperature below *T*_C_ in a 2D superconducting strip^37^, and the finite size of the sample may limit the low temperature divergence of the coherence length of the BKT transition leading to a vanishing resistive tail at low temperature^38^.

On the other hand, non percolating superconducting phases can display saturating non-zero resistance at zero temperature in amorphous systems^39^, or systems with inhomogeneities at low carrier densities^40^.

As they are produced by exfoliation, our samples are single crystals with low defect density as shown by the measured mobilities. However, the saturation resistivity shown by the 11 nm sample is too high to be explained by vortices or finite size effects alone. The space charge layer in the amorphous glass substrate may also display inhomogeneities at the nanometre scale. The resulting doping inside the crystalline MoS_2_, however, should be smoothed out by Coulomb screening, especially at high doping. Finally, the non-zero resistance could be attributed to some other extrinsic factor arising from our unique method. Preliminary measurements on a monolayer high critical temperature superconductor sample show that zero resistance superconductivity is attained in this sample under similar conditions, putting to doubt this last possibility. The saturation resistivity of the 11 nm sample even at the highest carrier densities is thus intriguing and will require further investigation.

The independence of *T*_C_ on carrier density (Fig. 7) is worth noting and different from the results reported in ref. 15. However, results similar to ours have recently been reported in ionic liquid doped WS_2_ (ref. 41). BCS superconductivity and the corresponding *T*_C_ depend on phonon frequencies, electron–phonon coupling and the density of states (DOS) at the Fermi level. In few-layer MoS_2_, the variations in the DOS with electron doping arise essentially from the particular variation of the conduction band which has two minima, at the K point and the Q point in the Brillouin zone^29^. The relative positions of these vary with the number of layers and the DOS changes in a step-like manner when the Fermi level crosses these minima. This could also account for differences in the superconductivity found in the 2 and 11 nm sample.

In conclusion, we have shown that very high doping densities that are required to trigger superconductivity can be reached by space charge doping using a simple and robust configuration of few-layer samples on glass substrates. This doping is reversible and applicable without risks of sample deterioration or electrochemical contamination which limit and complicate the use of other methods. It is also fully compatible with the anodic bonding method for sample fabrication making it a very competitive and efficient solution for such applications. We illustrate this by measuring the insulator–metal and superconducting transitions in few-layer MoS_2_. We find incomplete superconductivity independent of doping in a wide carrier density range but sensitive to confinement in ultra-thin samples. These findings provide a means for future studies in a variety of materials.

## Methods

### Sample preparation

Few-layer samples of MoS_2_ were made by the anodic bonding method^18^,^19^,^20^. Precursor flakes were carefully peeled off from natural molybdenite crystals and deposited on a 0.5 mm thick glass substrate (either standard soda-lime, or borosilicate Schott D263T). Substrate and precursor were put between two electrodes and heated between 100 and 200 °C to activate ionic mobility in the glass. Then a high voltage was applied between the two electrodes to attract sodium ions in the glass away from the interface with the precursor. After few minutes, the precursor is electrostatically stuck to the glass and the voltage can be removed. Using adhesive tape, the upper layers of the precursor can be mechanically exfoliated, leaving large area few-layer MoS_2_ on the glass substrate. The thickness of the samples were evaluated by optical contrast, Raman spectroscopy^42^ and confirmed by AFM topography measurements.

### Measurement details

Electrical contacts were made on selected MoS_2_ layers in Hall bar geometries by standard electron beam lithography techniques, followed by thermal evaporation of Cr (5 nm) and Au (70 nm) and liftoff. See Supplementary Fig. 4 for sample pictures. The backside of the glass substrate was then glued by silver epoxy to a gold electrode evaporated on top of an insulating MgO substrate to act as a back gate. Four point resistivity and Hall measurements were made in a high vacuum (*P*<10^−6^ mbar) Oxford He-flow cryostat with a minimum temperature of 2.8 K and maximum 420 K. A resistive electromagnet was used to apply magnetic fields up to 2 T perpendicular to the sample.

## Additional information

**How to cite this article:** Biscaras, J. *et al*. Onset of two-dimensional superconductivity in space charge doped few-layer molybdenum disulfide. *Nat. Commun.* 6:8826 doi: 10.1038/ncomms9826 (2015).

## Supplementary Material

Supplementary Information Supplementary Figures 1-4, Supplementary Notes 1-2 and Supplementary References

## Figures and Tables

**Figure 1:**
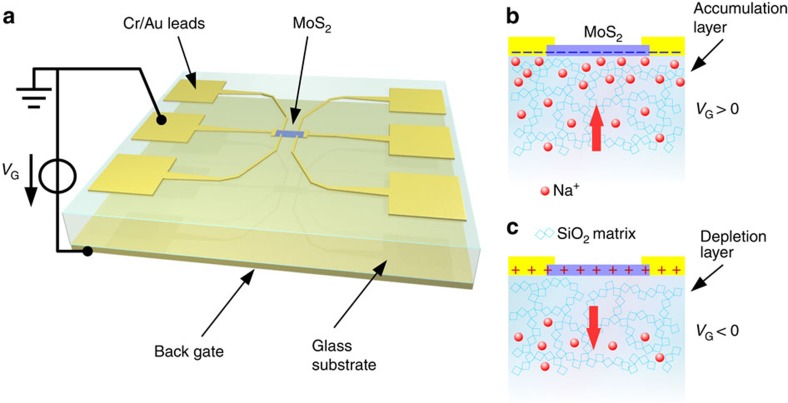
Space charge doping configuration. (**a**) Schematic 3D view of the space charge doping set-up with few-layer MoS_2_ contacted in a Hall bar pattern on a back-gated glass substrate. (**b**,**c**), Schematic view of the cross section of the MoS_2_ /glass substrate interface showing the creation of an accumulation (resp. depletion) layer by the movement of sodium ions under the applied electric field (red arrow).
