# Supplementary material for: Onset of two-dimensional superconductivity in space charge doped few-layer molybdenum disulfide
Source: Nat Commun. 2015 Nov 3;6:8826. doi: 10.1038/ncomms9826 (PMC4667624; doi:10.1038/ncomms9826)
Supplement: Supplementary Information — Supplementary Figures 1-4, Supplementary Notes 1-2 and Supplementary References [file ncomms9826-s1.pdf]

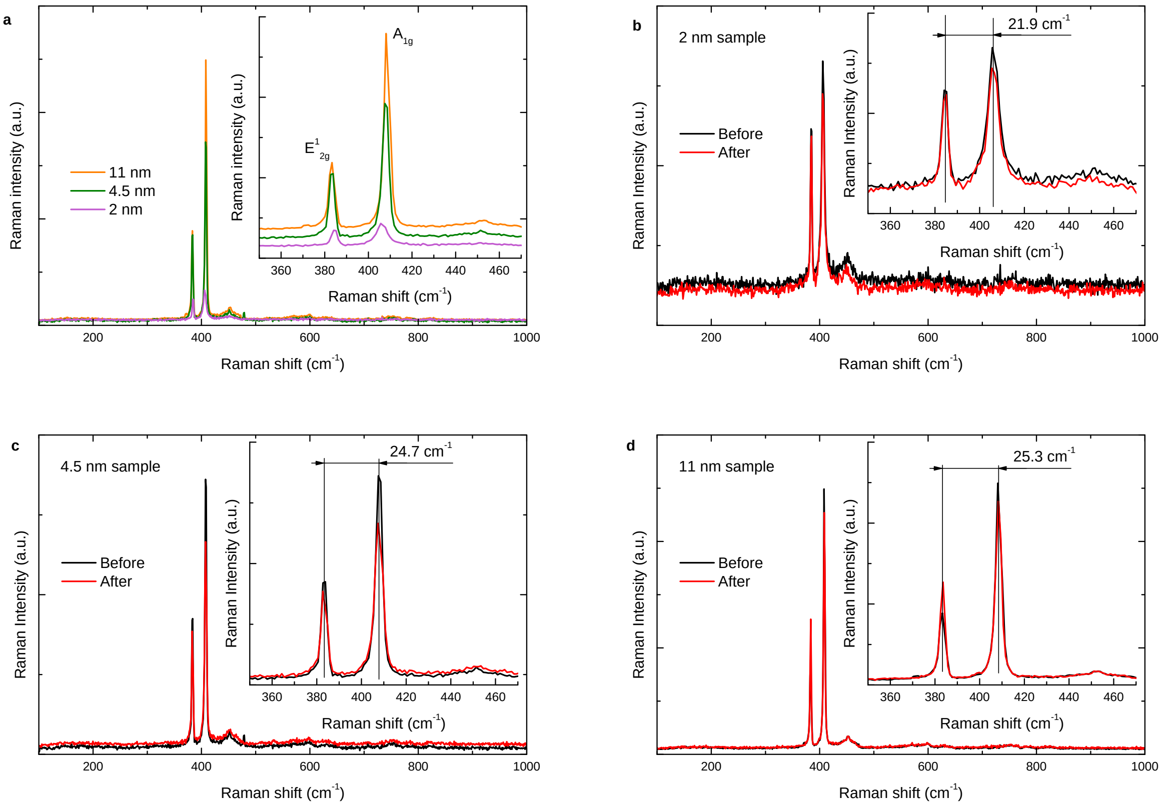

Supplementary Figure 1: Raman spectroscopy. a, Raman spectra of the three samples before measurement. **Inset :** zoom on the E^1_{2g} and A_{1g} lines. An offset was added for clarity. **b, c, d,** Raman spectra of each sample before and after doping and transport measurements.

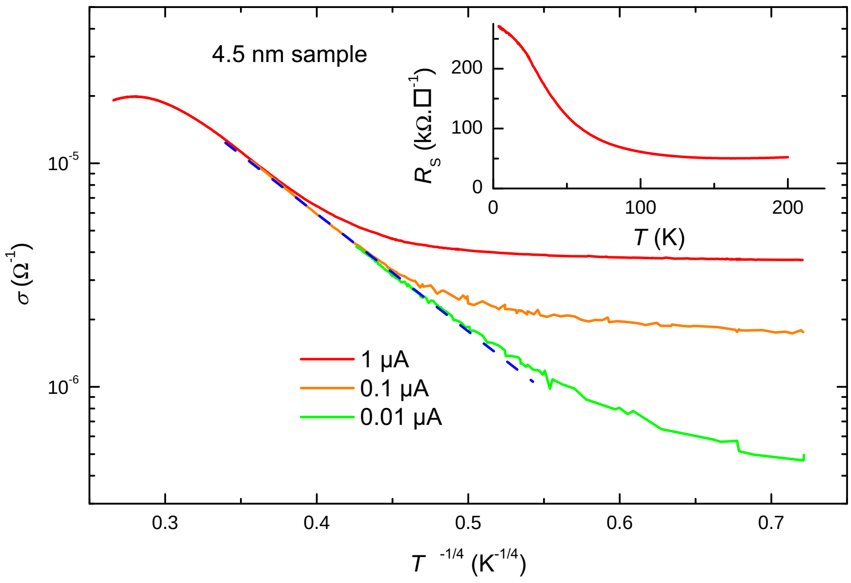

Supplementary Figure 2: 3D Variable Range Hopping. Conductivity of the 4.5 nm sample in the insulating regime as a function of $T^{-1/4}$ showing 3D Mott-Variable Range Hopping (M-VRH) behaviour. Inset : sheet resistance as a function of temperature.

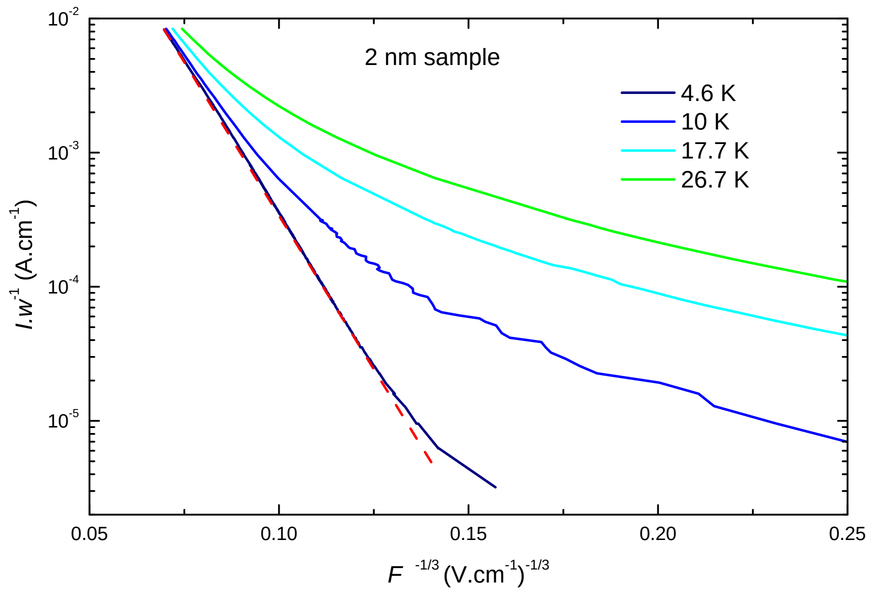

Supplementary Figure 3: 2D Variable Range Hopping. Current by unit width (w) of the 2 nm sample as a function of electric field to the power (-1/3) at different temperatures showing 2D M-VRH.

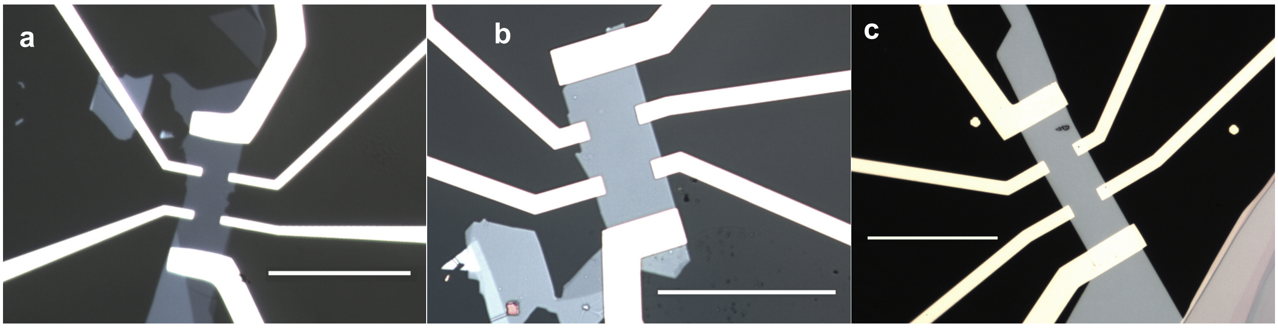

Supplementary Figure 4: Optical micrographs of the three samples with electrical contacts. The thickness of each sample has been measured by atomic force microscopy (AFM). **a,** 2 nm thick sample, scale bar 20 μm. **b,** 4.5 nm sample, scale bar 50 μm. **c,** 11 nm sample, scale bar 50 μm. Note that the picture of this sample was taken with reduced illumination power to avoid saturation and hence appears darker.

Supplementary Note 1. Raman spectroscopy before and after transport measurements

Raman spectra of the three samples were measured before and after space charge doping measurements. We used an Xplora Raman microscope (Horiba Jobin-Yvon) with 2400 lines/mm grating, $\sim$ 0.1 mW 532 nm laser illumination and a 100X microscope objective. As shown in Supplementary Figure 1 all samples display unaltered Raman spectra after the measurements. If there was chemical alteration of the material this would be seen in the post-measurement Raman spectra in these few-layer samples. Degradation through ambient pollution is clearly seen in Raman spectra of 2D samples of III-VI layered semi-conductors for example [1].The question is if crystalline quality alteration through defects would be seen. Even though there is no dedicated defect mode, Raman lines in MoS_2 are altered by the presence of defects through an increase in linewidths as in most other materials [2]. We see strictly no such change in our samples.

The energy difference between the two Raman active modes E^1_{2g} and A_{1g} varies monotonically with the number of layers and has been proposed as a measure of MoS_2 thickness for very thin samples [3–5]. This energy difference for the 2 nm sample is compatible with bilayer samples reported in literature.

Supplementary Note 2. Variable range hopping in the insulating regime

As shown in the paper, space charge doping gives access to an extended range of carrier density and to explore both sides of the metal-insulator transition. On the insulating side of the transition (*i.e.* at low carrier density), the resistivity of the sample increases dramatically at low temperature, and may become non-Ohmic. This insulating behaviour can be analysed to get informations about properties like dimensionality or the conduction mechanism.

At a carrier density of 4.5×10^{12} cm^{-2} the 4.5 nm sample displayed insulating behaviour reaching 270 $\mathrm{k}\Omega.\square^{-1}$ (measured with a current of 1 μA) at 4 K. The temperature behaviour is well described by variable range hopping (VRH) as shown in Supplementary Figure 2. As the name implies, VRH describes conductivity in insulating samples mediated by thermal or electric field activation of carrier hopping between localized states near the Fermi level. VRH manifests itself in the conductivity at low electric fields as a function of temperature in the form:

$$\sigma(T) = \sigma_0 \exp\left(-\frac{T_0}{T}\right)^{1/p} \tag{1}$$

where p is either $1/(D+1)$ in a D dimensional system with negligible Coulomb interaction between charged localized states (Mott-VRH [6]) or 1/2 in all dimensions in systems with substantial Coulomb gap (Efros Shklovskii-VRH [7]). As shown in Supplementary Figure 2 the 4.5 nm sample at low carrier density displays Mott-VRH conductivity with $p = 4$ characteristic of 3D transport.

At high electric field and low temperature, the field activation of conductivity becomes dominant over thermal activation, thus the current density becomes temperature independent and behaves as:

$$j \sim \exp\left(-\frac{F_0}{F}\right)^{1/p} \tag{2}$$

with the same p as above, and F being the electric field.

As shown in Supplementary Figure 3, the high electric field dependence of conductivity in the 2 nm sample display Mott-VRH with $p = 3$ characteristic of 2D transport.

As mentioned in the literature[8], it is difficult to distinguish between the possible exponents of VRH. A linear dependence of the conductivity with $T^{-1/4}$ will also appear nearly linear with $T^{-1/3}$ or $T^{-1/2}$. Accordingly we have shown here the results that present the most extended linear behaviour in temperature or electric field.

Supplementary References.

[1] Chen, Z., Biscaras, J. & Shukla, A. A high performance graphene/few-layer InSe photo-detector. *Nanoscale* **7**, 5981–5986 (2015).

[2] Mignuzzi, S. *et al.* Effect of disorder on raman scattering of single-layer MoS_2. *Phys. Rev. B* **91**, 195411 (2015).

[3] Lee, C. *et al.* Anomalous lattice vibrations of single- and few-layer MoS_2. *ACS Nano* **4**, 2695–2700 (2010).

[4] Late, D. J., Liu, B., Matte, H. S. S. R., Rao, C. N. R. & Dravid, V. P. Rapid characterization of ultrathin layers of chalcogenides on SiO_2/Si substrates. *Adv. Func. Mater.* **22**, 1894–1905 (2012).

[5] Boukhicha, M., Calandra, M., Measson, M.-A., Lancry, O. & Shukla, A. Anharmonic phonons in few-layer MoS_2: Raman spectroscopy of ultralow energy compression and shear modes. *Phys. Rev. B* **87**, 195316 (2013).

[6] Mott, N., Pepper, M., Pollitt, S., Wallis, R. H. & Adkins, C. J. The anderson transition. *Proc. R. Soc. A* **345**, 169–205 (1975).

[7] Efros, A. L. & Shklovskii, B. I. Coulomb gap and low temperature conductivity of disordered systems. *J. Phys. C: Solid State Phys.* **8**, L49 (1975).

[8] Joung, D. & Khondaker, S. Efros-shklovskii variable-range hopping in reduced graphene oxide sheets of varying carbon sp^2 fraction. *Phys. Rev. B* **86**, 235423 (2012).
